# *N*-Phenyl-6-(1*H*-pyrazol-1-yl)pyridazin-3-amine

**DOI:** 10.1107/S1600536810016697

**Published:** 2010-05-12

**Authors:** Abdul Qayyum Ather, M. Nawaz Tahir, Misbahul Ain Khan, Muhammad Makshoof Athar

**Affiliations:** a Department of Chemistry, Islamia University, Bahawalpur, Pakistan; b Applied Chemistry Research Center, PCSIR Laboratories complex, Lahore 54600, Pakistan; c Department of Physics, University of Sargodha, Sargodha, Pakistan; d Institute of Chemistry, University of the Punjab, Lahore, Pakistan

## Abstract

The mol­ecule of title compound, C_13_H_11_N_5_, is essentially planar (r.m.s. deviation = 0.0440 Å) and an intra­molecular C—H⋯N hydrogen bond generates an *S*(6) motif. In the crystal, mol­ecules are connected into chains by inter­molecular N—H⋯N and C—H⋯N hydrogen bonds. In addition, π–π stacking inter­actions are observed between the pyrazole and pyridazine rings [inter­planar distance = 3.6859 (10) Å].

## Related literature

For a related structure, see: Ather *et al.* (2009 ▶). For graph-set notation, see: Bernstein *et al.* (1995 ▶).
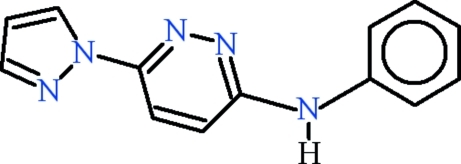


## Experimental

### 

#### Crystal data

C_13_H_11_N_5_
*M* *_r_* = 237.27
Orthorhombic, 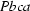

*a* = 11.3533 (7) Å
*b* = 9.4214 (5) Å
*c* = 21.6603 (14) Å
*V* = 2316.9 (2) Å^3^
*Z* = 8
Mo *K*α radiation
μ = 0.09 mm^−1^
*T* = 296 K
0.30 × 0.22 × 0.18 mm

#### Data collection

Bruker Kappa APEXII CCD diffractometer
Absorption correction: multi-scan (*SADABS*; Bruker, 2005 ▶) *T*_min_ = 0.982, *T*_max_ = 0.988
10085 measured reflections
2754 independent reflections
1571 reflections with *I* > 2σ(*I*)
*R*_int_ = 0.045

#### Refinement

*R*[*F* ^2^ > 2σ(*F* ^2^)] = 0.045
*wR*(*F* ^2^) = 0.119
*S* = 0.99
2754 reflections
163 parameters
H-atom parameters constrained
Δρ_max_ = 0.13 e Å^−3^
Δρ_min_ = −0.15 e Å^−3^

### 

Data collection: *APEX2* (Bruker, 2007 ▶); cell refinement: *SAINT* (Bruker, 2007 ▶); data reduction: *SAINT*; program(s) used to solve structure: *SHELXS97* (Sheldrick, 2008 ▶); program(s) used to refine structure: *SHELXL97* (Sheldrick, 2008 ▶); molecular graphics: *ORTEP-3 for Windows* (Farrugia, 1997 ▶) and *PLATON* (Spek, 2009 ▶); software used to prepare material for publication: *WinGX* (Farrugia, 1999 ▶) and *PLATON*.

## Supplementary Material

Crystal structure: contains datablocks global, I. DOI: 10.1107/S1600536810016697/gk2271sup1.cif

Structure factors: contains datablocks I. DOI: 10.1107/S1600536810016697/gk2271Isup2.hkl

Additional supplementary materials:  crystallographic information; 3D view; checkCIF report

## Figures and Tables

**Table 1:** Hydrogen-bond geometry (Å, °)

*D*—H⋯*A*	*D*—H	H⋯*A*	*D*⋯*A*	*D*—H⋯*A*
N1—H1⋯N5^i^	0.86	2.22	3.071 (2)	173
C6—H6⋯N2	0.93	2.37	2.966 (3)	122
C8—H8⋯N3^ii^	0.93	2.60	3.265 (2)	129

Symmetry codes: (i) 
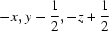
; (ii) 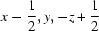.

**Fig. 1.**
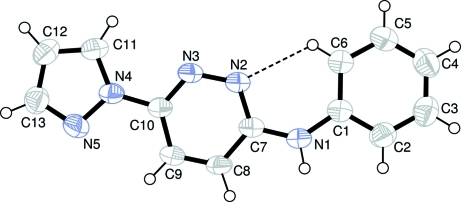
View of the title compound with the atom numbering scheme. The displacement ellipsoids are drawn at the 50% probability level. H-atoms are shown as small spheres of arbitrary radii. The dotted line indicates the intramolecular hydrogen bond.

**Fig. 2.**
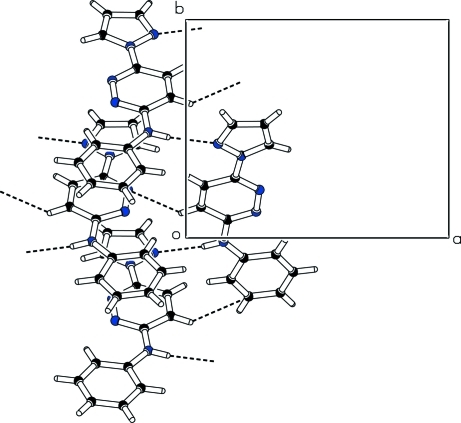
Packing diagram of the title compound (PLATON: Spek, 2009) showing that infinite polymeric chains extend along the b-axis.

## supplementary crystallographic information

### Comment

In continuation of our studies on pyrazolylpyridazine derivatives (Ather *et al.*, 2009), the structure of title compound ( Fig. 1) is reported here.

The title compound contains pyrazole, pyridazine and benzene rings. The r. m. s. deviation of 0.044 Å shows that the molecule of title compound is essentially planar. There exist S(6) ring motif (Bernstein *et al.*, 1995) due to C–H···N intramolecular H-bonding (Fig. 1). The molecules are stabilized in the form of infinite polymeric chains due to intermolecular H-bondings (Table 1) extending along the crystallographic b-axis (Fig. 2). The π–π interactions between the pyrazol and pyridazine ring are present at a distance of 3.6859 (10) Å.

### Experimental

3-Chloro-6-(1*H*-pyrazole-1-yl)pyridazine (1 g, 5.5 mmol) was dissolved in xylene (15 ml). Aniline (0.516 g, 5.5 mmol) was added to the solution and refluxed for 12 h. The reaction was monitored by TLC. After the completion, the reaction mixture was concentrated under vacuum. Distilled water (50 ml) was added to the resulting concentrated mixture, which give rise to precipitate. The filtered precipitate was dried and recrystallized from chloroform to obtain the title compound (I).

### Crystal data

**Table:**

C_13_H_11_N_5_	*F*(000) = 992
*M**_r_* = 237.27	*D*_x_ = 1.360 Mg m^−^^3^
Orthorhombic, *P**b**c**a*	Mo *K*α radiation, λ = 0.71073 Å
Hall symbol: -P 2ac 2ab	Cell parameters from 2920 reflections
*a* = 11.3533 (7) Å	θ = 2.6–27.9°
*b* = 9.4214 (5) Å	µ = 0.09 mm^−^^1^
*c* = 21.6603 (14) Å	*T* = 296 K
*V* = 2316.9 (2) Å^3^	Prismatic, pale brown
*Z* = 8	0.30 × 0.22 × 0.18 mm

### Data collection

**Table:**

Bruker Kappa APEXII CCD diffractometer	2754 independent reflections
Radiation source: fine-focus sealed tube	1571 reflections with *I* > 2σ(*I*)
graphite	*R*_int_ = 0.045
Detector resolution: 7.50 pixels mm^-1^	θ_max_ = 27.9°, θ_min_ = 2.6°
ω scan	*h* = −14→14
Absorption correction: multi-scan (*SADABS*; Bruker, 2005)	*k* = −12→8
*T*_min_ = 0.982, *T*_max_ = 0.988	*l* = −17→28
10085 measured reflections	

### Refinement

**Table:**

Refinement on *F*^2^	Primary atom site location: structure-invariant direct methods
Least-squares matrix: full	Secondary atom site location: difference Fourier map
*R*[*F*^2^ > 2σ(*F*^2^)] = 0.045	Hydrogen site location: inferred from neighbouring sites
*wR*(*F*^2^) = 0.119	H-atom parameters constrained
*S* = 0.99	*w* = 1/[σ^2^(*F*_o_^2^) + (0.0546*P*)^2^ + 0.0495*P*] where *P* = (*F*_o_^2^ + 2*F*_c_^2^)/3
2754 reflections	(Δ/σ)_max_ < 0.001
163 parameters	Δρ_max_ = 0.13 e Å^−^^3^
0 restraints	Δρ_min_ = −0.15 e Å^−^^3^

### Special details

**Table:**

Geometry. All esds (except the esd in the dihedral angle between two l.s. planes) are estimated using the full covariance matrix. The cell esds are taken into account individually in the estimation of esds in distances, angles and torsion angles; correlations between esds in cell parameters are only used when they are defined by crystal symmetry. An approximate (isotropic) treatment of cell esds is used for estimating esds involving l.s. planes.
Refinement. Refinement of F^2^ against ALL reflections. The weighted R-factor wR and goodness of fit S are based on F^2^, conventional R-factors R are based on F, with F set to zero for negative F^2^. The threshold expression of F^2^ > 2sigma(F^2^) is used only for calculating R-factors(gt) etc. and is not relevant to the choice of reflections for refinement. R-factors based on F^2^ are statistically about twice as large as those based on F, and R- factors based on ALL data will be even larger.

### Fractional atomic coordinates and isotropic or equivalent isotropic displacement parameters (Å^2^)

**Table:**

	*x*	*y*	*z*	*U*_iso_*/*U*_eq_	
C1	0.22072 (14)	−0.09114 (16)	0.11333 (7)	0.0450 (4)	
C2	0.17051 (16)	−0.18861 (17)	0.07303 (8)	0.0588 (5)	
H2	0.0892	−0.2008	0.0729	0.071*	
C3	0.23913 (19)	−0.2677 (2)	0.03321 (9)	0.0685 (5)	
H3	0.2040	−0.3327	0.0066	0.082*	
C4	0.35859 (18)	−0.2505 (2)	0.03286 (9)	0.0676 (5)	
H4	0.4052	−0.3027	0.0059	0.081*	
C5	0.40882 (17)	−0.1552 (2)	0.07273 (9)	0.0694 (5)	
H5	0.4902	−0.1440	0.0727	0.083*	
C6	0.34133 (15)	−0.07516 (18)	0.11315 (9)	0.0579 (5)	
H6	0.3772	−0.0112	0.1399	0.069*	
C7	0.15904 (13)	0.08370 (16)	0.19601 (7)	0.0432 (4)	
C8	0.05780 (14)	0.14252 (16)	0.22366 (8)	0.0499 (4)	
H8	−0.0169	0.1139	0.2113	0.060*	
C9	0.07086 (14)	0.24049 (17)	0.26820 (8)	0.0499 (4)	
H9	0.0066	0.2824	0.2876	0.060*	
C10	0.18694 (13)	0.27585 (16)	0.28379 (7)	0.0422 (4)	
C11	0.31668 (15)	0.42876 (18)	0.34775 (9)	0.0561 (5)	
H11	0.3892	0.4055	0.3305	0.067*	
C12	0.29780 (17)	0.52176 (19)	0.39459 (9)	0.0608 (5)	
H12	0.3537	0.5748	0.4159	0.073*	
C13	0.17754 (17)	0.52036 (18)	0.40374 (8)	0.0596 (5)	
H13	0.1392	0.5750	0.4333	0.072*	
N1	0.14245 (11)	−0.01684 (14)	0.15110 (6)	0.0493 (4)	
H1	0.0698	−0.0383	0.1450	0.059*	
N2	0.26728 (11)	0.12101 (13)	0.21296 (6)	0.0473 (4)	
N3	0.27965 (11)	0.22007 (14)	0.25801 (6)	0.0468 (3)	
N4	0.21109 (11)	0.37648 (13)	0.33089 (6)	0.0452 (3)	
N5	0.12287 (12)	0.43206 (15)	0.36555 (7)	0.0558 (4)	

### Atomic displacement parameters (Å^2^)

**Table:**

	*U*^11^	*U*^22^	*U*^33^	*U*^12^	*U*^13^	*U*^23^
C1	0.0436 (9)	0.0463 (9)	0.0452 (9)	0.0013 (7)	−0.0012 (8)	0.0076 (8)
C2	0.0529 (10)	0.0656 (11)	0.0579 (11)	−0.0040 (9)	−0.0040 (9)	−0.0029 (10)
C3	0.0790 (14)	0.0682 (12)	0.0583 (12)	−0.0014 (11)	−0.0065 (11)	−0.0114 (10)
C4	0.0691 (14)	0.0739 (13)	0.0599 (12)	0.0118 (11)	0.0068 (10)	−0.0065 (10)
C5	0.0490 (11)	0.0843 (13)	0.0748 (13)	0.0052 (10)	0.0041 (10)	−0.0132 (12)
C6	0.0465 (10)	0.0645 (11)	0.0627 (12)	−0.0005 (9)	0.0009 (9)	−0.0095 (9)
C7	0.0336 (8)	0.0461 (9)	0.0499 (9)	−0.0005 (7)	−0.0016 (7)	0.0067 (8)
C8	0.0290 (8)	0.0585 (10)	0.0621 (11)	−0.0005 (7)	−0.0047 (8)	−0.0022 (9)
C9	0.0313 (8)	0.0592 (10)	0.0593 (11)	0.0049 (7)	0.0002 (8)	−0.0038 (9)
C10	0.0340 (8)	0.0457 (9)	0.0468 (9)	0.0011 (7)	−0.0031 (7)	0.0051 (8)
C11	0.0400 (9)	0.0635 (11)	0.0648 (12)	−0.0036 (8)	−0.0072 (8)	−0.0002 (10)
C12	0.0606 (12)	0.0602 (11)	0.0617 (12)	−0.0044 (9)	−0.0155 (10)	−0.0052 (10)
C13	0.0632 (12)	0.0617 (11)	0.0540 (11)	0.0092 (9)	−0.0080 (10)	−0.0076 (9)
N1	0.0329 (7)	0.0563 (8)	0.0585 (9)	−0.0031 (6)	−0.0016 (6)	−0.0046 (7)
N2	0.0337 (7)	0.0552 (8)	0.0531 (8)	−0.0003 (6)	−0.0012 (6)	−0.0023 (7)
N3	0.0308 (7)	0.0557 (8)	0.0539 (8)	0.0006 (6)	−0.0015 (6)	−0.0007 (7)
N4	0.0347 (7)	0.0517 (8)	0.0491 (8)	0.0031 (6)	−0.0038 (6)	0.0038 (7)
N5	0.0435 (8)	0.0649 (9)	0.0589 (10)	0.0100 (7)	0.0008 (7)	−0.0042 (8)

### Geometric parameters (Å, °)

**Table:**

C1—C6	1.378 (2)	C8—H8	0.9300
C1—C2	1.389 (2)	C9—C10	1.401 (2)
C1—N1	1.3961 (19)	C9—H9	0.9300
C2—C3	1.381 (2)	C10—N3	1.3022 (19)
C2—H2	0.9300	C10—N4	1.4194 (19)
C3—C4	1.366 (3)	C11—N4	1.3465 (19)
C3—H3	0.9300	C11—C12	1.358 (2)
C4—C5	1.370 (2)	C11—H11	0.9300
C4—H4	0.9300	C12—C13	1.380 (3)
C5—C6	1.387 (2)	C12—H12	0.9300
C5—H5	0.9300	C13—N5	1.327 (2)
C6—H6	0.9300	C13—H13	0.9300
C7—N2	1.3300 (18)	N1—H1	0.8600
C7—N1	1.371 (2)	N2—N3	1.3574 (17)
C7—C8	1.410 (2)	N4—N5	1.3570 (17)
C8—C9	1.343 (2)		
			
C6—C1—C2	118.58 (16)	C8—C9—C10	116.13 (15)
C6—C1—N1	125.41 (15)	C8—C9—H9	121.9
C2—C1—N1	116.01 (15)	C10—C9—H9	121.9
C3—C2—C1	121.18 (18)	N3—C10—C9	124.14 (15)
C3—C2—H2	119.4	N3—C10—N4	114.93 (13)
C1—C2—H2	119.4	C9—C10—N4	120.92 (14)
C4—C3—C2	119.97 (18)	N4—C11—C12	107.35 (16)
C4—C3—H3	120.0	N4—C11—H11	126.3
C2—C3—H3	120.0	C12—C11—H11	126.3
C3—C4—C5	119.20 (18)	C11—C12—C13	104.92 (16)
C3—C4—H4	120.4	C11—C12—H12	127.5
C5—C4—H4	120.4	C13—C12—H12	127.5
C4—C5—C6	121.63 (18)	N5—C13—C12	112.29 (16)
C4—C5—H5	119.2	N5—C13—H13	123.9
C6—C5—H5	119.2	C12—C13—H13	123.9
C1—C6—C5	119.44 (17)	C7—N1—C1	132.45 (13)
C1—C6—H6	120.3	C7—N1—H1	113.8
C5—C6—H6	120.3	C1—N1—H1	113.8
N2—C7—N1	120.37 (14)	C7—N2—N3	118.41 (13)
N2—C7—C8	122.16 (15)	C10—N3—N2	120.13 (13)
N1—C7—C8	117.46 (14)	C11—N4—N5	111.46 (14)
C9—C8—C7	119.03 (15)	C11—N4—C10	127.68 (14)
C9—C8—H8	120.5	N5—N4—C10	120.86 (13)
C7—C8—H8	120.5	C13—N5—N4	103.98 (14)
			
C6—C1—C2—C3	−0.4 (2)	C6—C1—N1—C7	−1.0 (3)
N1—C1—C2—C3	179.63 (15)	C2—C1—N1—C7	178.96 (16)
C1—C2—C3—C4	−0.2 (3)	N1—C7—N2—N3	−179.41 (13)
C2—C3—C4—C5	0.6 (3)	C8—C7—N2—N3	−0.6 (2)
C3—C4—C5—C6	−0.5 (3)	C9—C10—N3—N2	0.0 (2)
C2—C1—C6—C5	0.5 (3)	N4—C10—N3—N2	179.08 (12)
N1—C1—C6—C5	−179.48 (15)	C7—N2—N3—C10	0.3 (2)
C4—C5—C6—C1	−0.1 (3)	C12—C11—N4—N5	−0.26 (19)
N2—C7—C8—C9	0.6 (2)	C12—C11—N4—C10	179.96 (14)
N1—C7—C8—C9	179.46 (14)	N3—C10—N4—C11	6.3 (2)
C7—C8—C9—C10	−0.3 (2)	C9—C10—N4—C11	−174.63 (16)
C8—C9—C10—N3	0.0 (2)	N3—C10—N4—N5	−173.47 (13)
C8—C9—C10—N4	−179.01 (13)	C9—C10—N4—N5	5.6 (2)
N4—C11—C12—C13	0.03 (19)	C12—C13—N5—N4	−0.37 (19)
C11—C12—C13—N5	0.2 (2)	C11—N4—N5—C13	0.39 (18)
N2—C7—N1—C1	−4.1 (3)	C10—N4—N5—C13	−179.82 (14)
C8—C7—N1—C1	177.09 (15)		

### Hydrogen-bond geometry (Å, °)

**Table:**

*D*—H···*A*	*D*—H	H···*A*	*D*···*A*	*D*—H···*A*
N1—H1···N5^i^	0.86	2.22	3.071 (2)	173
C6—H6···N2	0.93	2.37	2.966 (3)	122
C8—H8···N3^ii^	0.93	2.60	3.265 (2)	129

Symmetry codes: (i) −*x*, *y*−1/2, −*z*+1/2; (ii) *x*−1/2, *y*, −*z*+1/2.
